# Anti-Thyroid Peroxidase/Anti-Thyroglobulin Antibody-Related Neurologic Disorder Responsive to Steroids Presenting with Pure Acute Onset Chorea

**DOI:** 10.5334/tohm.175

**Published:** 2020-07-08

**Authors:** Ritwik Ghosh, Subhankar Chatterjee, Souvik Dubey, Alak Pandit, Biman Kanti Ray, Julián Benito-León

**Affiliations:** 1 Department of General Medicine, Burdwan Medical College and Hospital, Burdwan, West Bengal, IN; 2 Department of General Medicine, Rajendra Institute of Medical Sciences, Ranchi, IN; 3 Department of Neuromedicine, Bangur institute of Neurosciences, Kolkata, IN; 4 Department of Neurology, University Hospital “12 de Octubre”, Madrid, ES; 5 Centro de Investigación Biomédica en Red sobre Enfermedades Neurodegenerativas (CIBERNED), Madrid, ES; 6 Department of Medicine, Universidad Complutense, Madrid, ES

**Keywords:** Anti-thyroid peroxidase/anti-thyroglobulin antibody-related neurologic disorders responsive to steroids, Anti-thyroid peroxidase antibodies, Anti-thyroglobulin antibodies, Autoimmune, Chorea, Movement Disorders, Review, Steroid-responsive encephalopathy associated with autoimmune thyroiditis

## Abstract

**Background:**

Pure acute onset chorea without encephalopathy has rarely been reported in anti-thyroid peroxidase (anti-TPO)/anti-thyroglobulin (anti-TG) antibody-related neurologic disorders responsive to steroids (ATANDS).

**Case report:**

We report a 16-year-old female who presented with acute chorea without encephalopathy. Anti-TPO antibodies were found to be strongly positive (>1200 IU/ml) along with anti-thyroglobulin and anti-thyroid stimulating hormone receptor antibodies. After pulse intravenous methylprednisolone therapy (1 g/day for five consecutive days), all the movements seized, and she was discharged with oral prednisolone 30 mg/day with gradual tapering over next three months. After one year of follow-up, she is stable, drug-free, and never had any other problems.

**Discussion:**

Anti-thyroid antibodies testing should be included in routine/conventional panel that is done for elucidating causes of chorea as ATANDS can be easily missed and is treatable with widely available, relatively low-cost drugs like steroids with a promising outcome.

In autoimmune thyroid disorders, albeit known to affect only 1% of the population, focal or sub-clinical autoimmune thyroid inflammation can be found in around 15% of biochemically euthyroid population [1,2,3]. Anti-thyroid peroxidase (anti-TPO) and anti-thyroglobulin (anti-TG) antibodies are considered diagnostic markers of autoimmune thyroid disorders [1]. Neurological manifestations associated with autoimmune thyroid disorders have been frequently under-documented in literature [4], being the most protean amongst these disorders Hashimoto’s encephalopathy [5,6,7]. Spectrum of this disorder an range from subtle behavioral/personality changes to movement disorders, seizures, dementia, encephalopathy, stroke, coma and death [5,6,7,8]. Patients can also present with movement disorders without encephalopathy and cognitive impairment [5,6,7]. There is no pathognomonic clinical, serological, biochemical, electrophysiological or imaging markers [5,6,7]. In addition, there are no good predictors of treatment response to steroids; [9] in fact, in a recent study [9], only 31% patients completely responded to these drugs. Similarly, in other studies, only 56% and 36% patients with suspected Hashimoto’s encephalopathy responded to steroids [10,11]. Despite this, response to steroids seems to be the only partially consistent feature of this disorder hence renamed as “steroid responsive encephalopathy associated with autoimmune thyroiditis” (SREAT) [12], but neither response to steroids nor association with thyroiditis is steadfast [13]. Termasarasab et al., [14] have recently proposed “anti-TPO/TG antibody-related neurologic disorders responsive to steroids (ATANDS)” to be the renamed entity that would include the complete spectrum. Reported movement disorders that have been associated with ATANDS can be either “encephalopathic” or “non-encephalopathic” [5,6,7,14].

We had treated a patient with pure acute chorea who rapidly improved with corticosteroids. Here, we describe the case with a complete report. We also provide a review of the literature, which was performed to collect and summarize the present state of knowledge on movement disorders associated with ATANDS.

## Case presentation

A 16-year-old female presented to the neurology outpatient department with complaint of acute onset involuntary weird and quirky movements of all four limbs for last four days, which were irregular, asymmetric, rapid, unpredictable, purposeless, jerky and flowing from distal to proximally and that disappeared completely during sleep. Her past medical history was unremarkable. No associated febrile episode, seizure, headache, visual disturbances, behavioral changes, personality changes, forgetfulness, attention problems, or self-care inadequacy were noted. She had no history of any drug intake for any disease or substance abuse in recent past. No history was suggestive of any connective tissue disorder or thyroid dysfunction. Nobody in family had any neurological disease. On completion of an unremarkable general survey, detailed neurological examination revealed generalized chorea involving all extremities (right > left) with classic Jack in the box tongue and Milkman’s grip signs. Precise and meticulous cognitive assessment failed to unveil any impairment. Neither motor weakness, nor sensory deficits, nor signs of meningeal irritation and cranial nerve deficits were noted. Slit lamp examination ruled out Kayser-Fleischer ring.

No cognitive domain seemed to be affected and family history was negative; therefore, Huntington’s disease, Huntington’s disease-like syndromes, dentatorubral-pallidoluysian atrophy, and deposition disorders were virtually excluded. Hence, working diagnosis kept was acute onset generalized chorea without cognitive impairment. Differentials considered were: 1) metabolic chorea, 2) rheumatic chorea, 3) dysthyroidism associated chorea, 4) autoimmune chorea, 5) vascular chorea, and 6) chorea gravidarum.

Complete hemogram, thyroid, liver, kidney functions, electrolytes, arterial blood gas analysis and HbA1C were normal. A urine beta human chorionic gonadotropin and abdominal ultrasound ruled out any pregnancy. Serologies for human immunodeficiency virus, hepatitis C, and hepatitis B were negative. 24 hours urinary copper and serum ceruloplasmin levels were within normal range. Echocardiography, serial anti-streptolysin O titers, and anti-DNase B antibodies levels ruled out possibility of Sydenham’s chorea. Anti-Nuclear Antibody (ANA) screening using HEp-2 cells, ANA profile, antiphospholipid antibodies, and antineutrophil cytoplasmic antibodies (cANCA and pANCA) were found to be negative. Autoantibodies directed against voltage gated potassium channel and anti-N-methyl-D-aspartate receptor antibodies were also negative. Magnetic resonance imaging of brain, electroencephalogram and cerebrospinal fluid analyses were otherwise normal.

Serum anti-TPO antibodies were found to be strongly positive (>1200 IU/ml) along with anti-TG and anti-thyroid stimulating hormone receptor antibodies. Patient was put on pulse intravenous methylprednisolone therapy (1 g/day for five consecutive days). All the movements seized and she was discharged with oral prednisolone 30 mg/day with gradual tapering over next three months. Tests were rerun with similar results, but anti-thyroid stimulating hormone receptor antibodies, which were within normal range this time around. At present, after one year of follow-up, she remains stable, drug-free and without any other problems.

## Discussion

ATANDS with associated movement disorders have been described previously (Table 1) [15,16,17,18,19,20,21,22,23,24,25,26,27,28,29,30,31,32,33,34,35,36,37,38,39,40,41,42,43,44,45,46,47,48,49,50]. We have reported a 16-year-old female with ATANDS who presented with acute pure chorea without encephalopathy. ATANDS presenting with chorea is exceedingly rare. For example, Miranda et al., [47] described a middle-aged female with acute onset rapidly worsening choreo-athetosis with dystonia and slurred speech which came out to be a case of ATANDS. Sharan A et al., [41] reported an aged female with ATANDS, who developed abrupt onset behavioral changes along with asymmetric florid chorea. Taurin G et al., [19] narrated behavioral abnormality with psychotic features along with bilateral and axial choreic movements in an elderly female. Our patient had no behavioral abnormalities or any other extrapyramidal or cerebellar features unlike those previously mentioned cases [19,41,47]. In all those cases steroid resulted in good yield alike our patient [19,41,47].

**Table 1:** Movement disorders associated with anti-TPO/TG antibody-related neurologic disorders responsive to steroids.

	Author and year of publication	Age/Sex	Type of movement disorder	Thyroid status	Anti-thyroid antibody	Neuroimaging	Treatment	Outcome

1.	Mehta AB et al., [15] 1981	17/F	Torsion dystonia	Primary hyperthyroidism/thyrotoxicosis	Anti-TG+ Anti-TPO+	No data available	Carbimazole and radioactive iodine	Euthyroid, dystonia was very mild with a slight tendency for torticollis and scoliosis to the right, and for the right outstretched arm to hyperpronate, provided drug compliant
2.	Javaid A and Hilton DD [16] 1988	15/F	Generalized choreo-athetosis	Primary hyperthyroidism	Anti-TG+ Anti-TPO+	No data available	Propranolol, carbimazole, tetrabenazine, chlorpromazine, and haloperidol	Refractory chorea. Chlorpromazine and tetrabenazine only partially suppressed it. At six months it was persisting. Haloperidol almost completely abolished chorea. It returned whenever she stopped taking haloperidol. Recurrence occurred 16 months after she first presented
3.	Baba M et al., [17] 1992	23/F	Hemichorea	Primary hyperthyroidism/Graves’ disease	Anti-TG+ Anti-TPO-	No changes	Metoprolol, thiamazole and chlorpromazine	Improved
4.	Hernández Echebarría LE et al., [18] 2000	41/F	Opsoclonus, myoclonus, and gait ataxia	Euthyroid→ subclinical hypothyroidism	Anti-TPO+	SPECT showed decreased perfusion in the left fronto-parietal region and in the right basal ganglia	Antibiotics, acyclovir and valproate followed by L-thyroxin and steroids	At one-year follow-up, CSF analysis, SPECT, and electroencephalogram were normal. Anti-TPO decreased. She remained well at the last visit, two years after the onset of neurologic symptoms.
5.	Taurin G et al., [19] 2002	77/F	Bilateral and axial choreic movements	Primary hypothyroidism	Anti-TG+ Anti-TPO+	Cortico-subcortical atrophy	L-thyroxin and oral prednisolone	With 60 mg/day of prednisolone, chorea disappeared and reappeared again; on increasing dose to 80 mg/d, it disappeared. At 3 weeks, the patient was clinically normal. No relapse during 8 months of follow-up
6.	Erickson JC et al., [20] 2002	34/M	Myorhythmia, myoclonus, and tremor	Primary hypothyroidism	Anti-TG+ Anti-TPO+	No changes	IVMP followed by oral prednisolone	Moderate improvement with residual mild cognitive impairment and subtle facial myorhythmia
7.	Erickson JC et al., [20] 2002	38/M	Palatal tremor	Euthyroid	Anti-TPO+ Anti-TG-	Venous anomaly in hypothalamus	IVMP followed by oral prednisolone	Moderate improvement in seizures, but cognitive impairment persisting
8.	Nagpal T and Pande S [21] 2004	52/F	Parkinsonism and myoclonus	Subclinical hypothyroidism	Anti-TPO+ Anti-TG-	Cerebral atrophy	IVMP followed by oral prednisolone, PLEX, and finally by oral prednisolone	No improvement with IVMP; significant improvement 10days after PLEX
9.	Loh LM et al., [22] 2005	40/M	Propriospinal or segmental myoclonus, Spasmodic truncal flexion	Primary hyperthyroid/Graves’ disease	TRAB+ Anti-TG+ TSI+ Anti-TPO-	No changes	Clonazepam and propylthiouracil	Euthyroidism established and symptoms improved
10.	Tan EK et al., [23] 2006	Middle aged/M	Bilateral postural hand tremor and task-specific dystonia-writer’s cramp	Primary hyperthyroidism/Graves’ disease	Anti-TG+	No changes	Carbimazole	Euthyroidism achieved and symptoms improved
11.	Guimaraes J et al., [24] 2007	60/M	Painful legs and moving toes syndrome, bradykinesia, and dystonia	Primary hypothyroidism	Anti-TG+ Anti-TPO+	Subcortical white matter lesions	Oral prednisolone	No improvement
12.	Tan EK et al., [25] 2008	50/F	Isolated orthostatic tremor	Primary hyperthyroidism/Graves’ disease	TRAB+ Anti-TG+ Anti-TPO+	No changes	carbimazole	Complete resolution
13.	Ku CR et al., [26] 2008	42/F	Generalized chorea	Primary hyperthyroidism/Graves’ disease	TRAB+ Anti-TG- Anti-TPO+	No changes	IVMP, propylthiouracil, propranolol, trihexyphenidyl, ropinirole, clonazepam and quetiapine	Improved
14.	Yu JH and Weng YM [27] 2009	17/F	Chorea	Primary hyperthyroidism/Graves’ disease	Anti-TPO+	SPECT revealed decreased perfusion to the right anterior temporal cortex	Propylthiouracil and propranolol	Complete resolution
15.	Broch L and Amthor KF [28] 2010	66/F	Myoclonus, tremor	Euthyroid	Anti-TPO++	No changes	Systemic steroids	Improved
16.	Salazar R et al., [29] 2012	59/M	Opsoclonus and gait ataxia	Euthyroid	Anti-TG+ Anti-TPO+	No changes	IVIG/IVMP	After three months of therapy with corticosteroids improved, but not with IVIG
17.	Liu MY et al., [30] 2012	75/M	Paroxysmal kinesigenic dyskinesia	Unknown	Anti-TPO+	No changes	IVMP followed by oral prednisone taper	Back to baseline in 20days
18.	Inoue K et al., [31] 2012	63/F	Micrography, parkinsonian gait and tremor	Euthyroid	Anti-TPO+	White matter ischemic changes	IVMP followed by oral prednisolone	Improved
19.	Ryan SA et al., [32] 2012	48/M	Myoclonus	Subclinical hypothyroidism	Anti-TPO++ Anti-TG-	No changes	Oral prednisolone	Improved
20.	Park J et al., [33] 2012	16/M	Asymmetric chorea	Primary hyperthyroidism/Graves’ disease	TRAB+ Anti-TG+	No changes	Propylthiouracil and propranolol	Improved
21.	Nakavachara P et al., [34] 2013	14/M	Choreo-athetosis	Primary hyperthyroidism/Graves’ disease	Anti-TPO+ Anti-TG+	No data available	Methimazole, propranolol, and IV potassium	Improved
22.	Kaminska A et al., [35] 2013	23/F	Hemi-chorea	Primary hyperthyroidism/Graves’ disease	TRAB+ Anti-TPO+	No changes	Thiamazole, prednisolone, haloperidol, thioridazine	Subsidence of symptoms
23.	Ghoreishi E et al., [36] 2013	32/M	Palatal myoclonus	Euthyroid	Anti-TG+ Anti-TPO+	Bilateral striatal hyperintensity on T2WI	Oral prednisolone	Improved
24.	Philip R et al., [37] 2014	18/M	Myoclonus and tremor	Primary hyperthyroidism	Anti-TPO+	Non-specific white matter changes and pituitary hyperplasia	IVMP followed by oral prednisolone and L-thyroxin	Significant improvement
25.	Saygi S et al., [38] 2014	12/M	Motor tics	Euthyroid	Anti-TPO++ Anti-TG+	No changes	Oral prednisolone	Improved
26.	Rozankovic PB et al., [39] 2015	27/F	Myoclonus-Dystonia and choreo-athetosis	Euthyroid	Anti-TPO+	No changes	IVMP followed by oral Prednisolone	Complete resolution
27.	Lee HJ et al., [40] 2015	30/M	Ocular flutter, limb and gait ataxia, myoclonus, and truncal titubation	Euthyroid	Anti-TG+ Anti-TPO-	No changes	IVMP followed by oral prednisolone	Improved
28.	Sharan A et al., [41] 2015	78/F	Choreiform movements	Euthyroid	Anti-TPO+	Atrophy	IVMP followed by oral prednisolone	Improved
29.	Sheetal SK et al., [42] 2016	66/M	Action-myoclonus, parkinsonism (corticobasal disease-variant-like)	Euthyroid	Anti-TPO+ Anti-TG-	Small right thalamic hematoma	IVMP followed by oral prednisolone	Improved
30.	Ramcharan K et al., [43] 2016	34/F	Bilateral hand postural tremor	Euthyroid	Anti-TG+ TRAB+ Anti-TPO-	No changes	Oral prednisolone	Improved
31.	Correia I et al., [44] 2016	61/F	Limb myoclonus	Primary hypothyroidism	Anti-TPO+	SPECT revealed hypoperfusion in frontal, temporal, and parietal regions with left predominance	L-thyroxin, IVMP followed by oral prednisolone and azathioprine	Resolution
32.	Kelly DM et al., [45] 2017	64/M	Abdominal tremor and abdominal wall dyskinesia	Primary hyperthyroidism	Anti-TPO+	No changes	Carbimazole and supportive	Complete resolution
33.	Keshavaraj A and Anpalagan J [46] 2018	23/F	Oro-lingual dyskinesia	Primary hyperthyroidism	Anti-TPO+ TRAB+	No changes	IVMP, carbimazole, and propranolol	Significant improvement
34.	Miranda M et al., [47] 2018	34/F	Choreo-athetosis, dystonia and ataxia	Euthyroid	Anti-TG+ Anti-TPO+	No changes	IVMP and IVIG	Complete resolution
35.	Miranda M et al., [47] 2018	61/M	Myoclonus-dystonia	Euthyroid	Anti-TPO+	No changes	IVMP, IVIG, PLEX and rituximab	Incomplete resolution
36.	Mohd Fauz NA et al., [48] 2019	50/F	Relapsing-remitting opsoclonus-myoclonus-ataxia syndrome	Subclinical hyperthyroidism	Anti-TG- Anti-TPO+	Lesions in cortical and subcortical regions, pons and midbrain	IVMP and PLEX	Improved
37.	Delhasse S et al., [49] 2019	60/F	Complex dyskinesia, high-amplitude myoclonic jerks, mild chorea, and postural tremor	Primary hyperthyroid/Graves’ disease	TRAB+ Anti-TG+ Anti-TPO+ Isotope scan+	No changes	Carbimazoleàpropylthiouracilàradio-active iodine	Complete resolution
38.	ShreeS et al., [50] 2020	60/F	Tremor, myoclonus and catatonia	Euthyroid	Anti-TPO+	No changes	IVMP followed by oral prednisolone.	Improved

F: female; M: male; Anti-TPO: anti-thyroid peroxidase antibody; Anti-TG: anti-thyroglobulin antibody; TSI: thyroid stimulating immunoglobulin; TRAB: TSH receptor antibody; CSF: cerebrospinal fluid; SPECT: Single-photon emission computed tomography; IV: intravenous; MP: methylprednisolone; IG: immunoglobulin; PLEX: plasma exchange; MRI: magnetic resonance imaging; ‘+’: high/positive titer; ‘–’: low/negative titer.

Etiopathogenetic factors for chorea are believed to be a) a hypersensitivity of dopaminergic receptors to dopamine due to a thyrotoxic state; [17,51] b) derangements in cerebral perfusion as reported in cases of acute onset chorea associated with other etiologies [27,52,53] and substantiated by single-photon emission computed tomography and positron emission tomography imaging; [54,55] c) autoimmune central nervous system vasculitis; [8,56] and d) anti-thyroid antibody mediated effects on neurons [7,57,58,59]. However, for neurological manifestations like chorea, anti-thyroid antibodies are extremely sensitive, but lack specificity [60]. And whether they are pathogenic or just a marker of the disease or just an epiphenomenon, remains elusive [6,7,60,61]. Presence of anti-thyroid antibodies in general population is well established even in absence of neurologic disorders and usually acting as a confounding factor in diagnosis [60]. Further, levels of anti-thyroid antibodies do not correlate well with disease severity and often persist in high levels after the treatment and clinical response [6,62,63].

In conclusion, our experience with the current case and our review of the literature strongly suggest that ATANDS/SREAT can rarely present with movement disorders alone and may be a definite separate entity. Anti-thyroid antibody testing should be included in routine/conventional panel that is done for elucidating causes of chorea as this disorder can be easily missed and is treatable with widely available, relatively low-cost drugs like steroids with a promising outcome.
